# Neural Effects of Auditory Distraction on Visual Attention in Schizophrenia

**DOI:** 10.1371/journal.pone.0060606

**Published:** 2013-04-01

**Authors:** Jason Smucny, Donald C. Rojas, Lindsay C. Eichman, Jason R. Tregellas

**Affiliations:** 1 Neuroscience Program, University of Colorado Anschutz Medical Campus, Aurora, Colorado, United States of America; 2 Department of Psychiatry, University of Colorado Anschutz Medical Campus, Aurora, Colorado, United States of America; 3 Research Service, Denver VA Medical Center, Denver, Colorado, United States of America; Cardiff University, United Kingdom

## Abstract

Sensory flooding, particularly during auditory stimulation, is a common problem for patients with schizophrenia. The functional consequences of this impairment during cross-modal attention tasks, however, are unclear. The purpose of this study was to examine how auditory distraction differentially affects task-associated response during visual attention in patients and healthy controls. To that end, 21 outpatients with schizophrenia and 23 healthy comparison subjects performed a visual attention task in the presence or absence of distracting, environmentally relevant “urban” noise while undergoing functional magnetic resonance imaging at 3T. The task had two conditions (difficult and easy); task-related neural activity was defined as difficult – easy. During task performance, a significant distraction (noise or silence) by group (patient or control) interaction was observed in the left dorsolateral prefrontal cortex, right hippocampus, left temporoparietal junction, and right fusiform gyrus, with patients showing relative hypoactivation during noise compared to controls. In patients, the ability to recruit the dorsolateral prefrontal cortex during the task in noise was negatively correlated with the effect of noise on reaction time. Clinically, the ability to recruit the fusiform gyrus during the task in noise was negatively correlated with SANS affective flattening score, and hippocampal recruitment during the task in noise was positively correlated with global functioning. In conclusion, schizophrenia may be associated with abnormalities in neural response during visual attention tasks in the presence of cross-modal noise distraction. These response differences may predict global functioning in the illness, and may serve as a biomarker for therapeutic development.

## Introduction

Development of neuroimaging biomarkers for cognitive symptoms of schizophrenia, including deficits in sustained and selective attention, remains a priority for neuropsychiatric research. Important issues in the selection of these biomarkers are relevance to the symptomatic presentation of the disorder as well as the presence of established neurobiological models that may underlie the cognitive phenotype.

In early behavioral investigations of schizophrenia, patients often complained of being unable to ignore distracting sounds in the environment [1]. This deficit has been hypothesized to reflect inhibitory dysfunction in brain areas important for sensory filtering, such as the hippocampus [2]–[6]. In support of this theory, patients show reduced gating of early (50 ms post-stimulus) event related potentials during repeated clicks [2], as well as increased hippocampal and dorsolateral prefrontal (DLPFC) response during passive listening to click trains [3] and environmental “urban noise” [4].

Sensory processing deficits are increasingly recognized as key contributors to cognitive dysfunction in schizophrenia [7], [8]. In particular, current hypotheses suggest that sensory filtering is an important mechanism by which to reduce the burden on executive systems during cognitively demanding tasks [8]–[11]. However, few neuroimaging studies have directly examined how sensory filtering deficits contribute to functional abnormalities in cortical areas during cognitive tasks in the illness. We have recently reported an inverse correlation between hippocampal activity during passive listening to urban noise and recruitment of the temporoparietal junction (TPJ) during an auditory tone discrimination task (with noise distraction) in patients, suggesting that hippocampal hyperactivity during noise may impair the ability of patients to engage task-relevant systems. In addition to passive listening, hippocampal hyperactivity in schizophrenia has been observed during other tasks requiring minimal or no cognitive load, such as fixating on a point [12], smooth pursuit eye movement [13], and resting state (a scan with no task) [14]. Hippocampal hyperactivity during low load may thus be a general mechanism by which, relative to controls, patients are less able to appropriately increase brain activity as task difficulty is increased, resulting in hypoactivation [15].

Our previous findings using the tone discrimination task suggest that distractors of the same sensory modality (auditory) may differentially affect neural response in patients. However, “real world” situations often involve cross-modal filtering – e.g. ignoring irrelevant noise while performing a visual task. Previous studies have demonstrated that patients are more behaviorally impaired than controls during both intramodal and cross-modal distractors [16], [17]. To our knowledge, however, no study has yet examined the functional neural correlates of cross-modal, auditory distraction on visual attention in schizophrenia.

The purpose of the present study was to use fMRI to examine neural response associated with environmental noise distraction in schizophrenia patients and healthy comparison subjects during a visual attention task. We hypothesized that patients would show altered neural response as task difficulty was increased in the hippocampus, DLPFC, TPJ, and fusiform gyrus, given previous studies that have shown abnormal activity in patients in these regions during passive listening [4] and/or selective attention tasks [15], [18], [19]. Based on previous findings showing hippocampal hyperactivity during passive listening to noise, we further postulated that relative to controls, patients would show increased response in the hippocampus during noise under easy, low-load conditions. We also hypothesized that as task difficulty was increased, hippocampal recruitment during noise would be correlated with Global Assessment of Function (GAF) score, based on previous work demonstrating an association between patient functioning and sensory filtering ability [20].

## Materials and Methods

### Participants

44 subjects participated in this study - 21 stable outpatients who met DSM-IV criteria for schizophrenia (7 women and 14 men; mean age = 46.9 years, SD = 12.4) and 23 healthy comparison subjects (10 women and 13 men; mean age = 39.4 years, SD = 12.3). Patients were recruited by referral from a University of Colorado psychiatrist and by other local clinicians and mental health professionals. No significant group differences in age or gender were observed. GAF, Brief Psychiatric Rating Scale (BPRS), and Scale for the Assessment of Negative Symptoms (SANS) scores were also collected in patients during diagnostic interviews. Of the 21 persons with schizophrenia, 20 were treated with atypical antipsychotics, and one with conventional antipsychotics. Subjects were compensated for participation. The Colorado Multiple Institution Review Board approved the study, and all participants provided written informed consent in accordance with the principles of the Declaration of Helsinki. All potential participants who declined to participate or otherwise did not participate were eligible for treatment (if applicable) and were not disadvantaged in any other way by not participating in the study.

### fMRI Methods

Studies were performed with a 3T GE Signa MR system using a standard quadrature head coil. Functional images were acquired with a gradient-echo T2* Blood Oxygenation Level Dependent (BOLD) contrast technique, with TR = 12650 ms (as a clustered volume acquisition of 2000 ms scanning, plus an additional 10650 ms silent interval), TE = 30 ms, FOV  = 220 mm^2^, 64^2^ matrix, 38 slices, 3 mm thick, 0.5 mm gap, angled parallel to the planum sphenoidale. Clustered volume acquisition was used because it minimizes the confounding effects of scanner noise on the auditory task, and improves sensitivity to the BOLD response during such tasks [21]. This acquisition method has been used previously to image brain activity in patients during multiple auditory sensory processing paradigms [3], [4], [15]. At the end of the session, one IR-EPI (TI = 505 ms) volume was acquired to improve spatial normalization.

Head motion was minimized with a VacFix head-conforming vacuum cushion (Par Scientific A/S, Odense, Denmark). Auditory stimuli were presented via MR-compatible headphones (Resonance Technology, Inc., CA, USA). Visual stimuli were presented via MR-compatible goggles (Resonance Technology, Inc, CA, USA). Motor responses were collected via a fiber optic response pad (Cedrus Corp, USA).

### fMRI Paradigm

fMR images were obtained while subjects performed the Sustained Attention to Response Task (SART) as described previously [22]. Subjects were shown single-digit numbers presented one-at-a-time, and instructed to press a button after every number except for the number “3,” in which case subjects were asked to withhold responding. Two task conditions were used that differed in difficulty [22]. In the easy (‘Ordered’) condition, single-digit numbers were presented sequentially (i.e. 1,2,3,4…). In the more difficult (‘Random’) condition, numbers were presented pseudo-randomly. The subject was asked to respond as quickly and accurately as possible to help induce attentiveness.

Task-relevant stimuli were presented as a block design, with ‘Ordered’ and ‘Random’ blocks pseudo-randomly interspersed throughout a session. A 2.3s identifier cue (i.e. Ordered or Random) was presented before the first block, as well as each time the block switched from Ordered to Random (or vice-versa). The length of each block was 12.65s. Blocks that were preceded by an instruction had 9 trials; blocks that were not preceded by an instruction had 11 trials, thus making each block equal in duration. Each trial consisted of a 250 ms stimulus (the single-digit number) followed by a 900 ms intertrial interval; during the intertrial interval a fixation cross was presented to orient the subject. Number font was pseudo-randomized (40, 72, 94, 100, 120 type) to increase the difference in feature detection processing requirements between the easy and difficult versions. Due to the predictability of the easy task, subjects may be able to correctly respond or withhold responding reflexively to the presence of any visual stimulus; however, the unpredictability of the more difficult task requires subjects to focus on specific stimulus features to a greater degree to make the appropriate response. Each session consisted of 56 blocks of trials and lasted for approximately 12 m. Baseline data were collected from a 37.95s fixation period at the beginning and end of each session, and two 12.65s fixation sessions near the middle. Subjects were given a brief practice session outside of the scanner to introduce them to the task parameters.

To determine the effect of noise distraction on the functional neuroanatomy of the task, previously developed 80 dB “urban noise” distractors (Tregellas et al. 2009) were presented during half of the blocks, in pseudorandom order. Noise was presented in the magnet through MR-compatible headphones (Resonance Technology, Inc.). The “urban noise” consisted of a mixture of audio clips, including segments of radio shows, classical music pieces, and background conversation [4]. Volumes of all of these elements were mixed so that no one element was readily identifiable. The subjective experience of the sound mixture was that of standing in a busy crowd of people, in which multiple conversations were occurring, with a low level of indistinguishable background music and other sounds one might experience in a busy urban setting.

### Behavioral Data Analysis

Performance measures for each task condition were: 1) percent commission errors, defined as the percent of incorrect responses on no-go trials, i.e. the percent of button presses following presentation of the number “3” (when no response was expected) 2) percent omission errors, defined as the percent of incorrect responses on go trials, i.e. the percent of omitted responses following presentation of numbers others than “3” (when responses were expected) and 3) reaction times during go trials.

Data for each of the three performance measures were analyzed using a repeated measures ANOVA, with group (patient vs control) as a between-subjects factor and condition (OrderedSilent, OrderedNoise, RandomSilent, RandomNoise) as a within-subjects factor. Behavioral data were unavailable for four patients due to technical issues in response collection. All behavioral data were analyzed using SPSS20 (IBM, NY, USA).

### fMRI Data Analysis

Data were analyzed with SPM8 (Wellcome Department of Imaging Neuroscience, London). Echo-planar images (EPI) from each subject were realigned to the first volume. The realigned images were then normalized to the Montreal Neurological Institute template using the unified segmentation algorithm [23] on the IR-EPI image and applying the estimated warp parameters to the coregistered EPI data. During normalization, data were resliced to a 3 mm^3^ voxel size. Finally, functional images were smoothed with an 8-mm FWHM Gaussian kernel. A 196s high-pass filter was applied to remove low-frequency fluctuation in the BOLD signal.

The hemodynamic response was modeled with a double gamma function, without temporal derivatives, using the general linear model in SPM8. To account for both within-group and within-subject variance, a random effects analysis was implemented. First-level task-associated contrast images were generated for the four task conditions, OrderedSilent, OrderedNoise, RandomSilent, and RandomNoise, with fixation periods as an implicit baseline. Parameter estimates for each individual's first level analysis (SPM contrast images) were entered into a second-level flexible factorial ANOVA in SPM8. Planned comparisons were evaluated with directional contrasts (SPM t-contrasts). For visualization purposes, statistical parametric maps were thresholded at p<0.01.

In this study, “task” related activity was defined as the effect of difficulty:
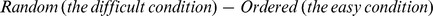



The effect of noise was defined as: 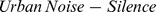



The primary contrast of interest analyzed task-related activity, using the diagnosis (patient vs control) X noise (noise vs silence) interaction: 
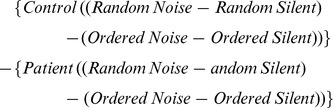



To compare the effect of noise in patients vs. controls under easy conditions, the following contrast was utilized: 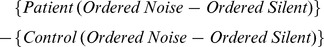



Behavioral correlations were analyzed using identical contrasts.

*A priori* hypotheses about response in four regions, the hippocampus, TPJ, and DLPFC, and fusiform gyrus, were examined. The hippocampal ROI consisted of a 10 mm sphere centered at x = 30, y = −15, z = −14, the location previously observed to show increased response during passive listening to noise in patients [4]. The DLPFC ROI was a 10 mm sphere centered at x = −39, y = 30, z = 39, the TPJ ROI was a 10 mm sphere centered at x = −60, y = −36, z = 24, and the fusiform ROI was a 10 mm sphere centered at x = 48, y = −74, z = −13. These locations have been previously observed to show abnormal response during selective attention in schizophrenia [18], [19], [24]. fMRI results were corrected for multiple comparisons with the Small Volume Correction (SVC), FWE-corrected p<0.05. Correlations with GAF, SANS, and BPRS scores were performed using peak values from within the defined ROIs. Bonferroni corrections were applied to SANS (threshold p = 0.0125) and BPRS subscores (threshold p = 0.0025).

## Results

### Behavioral Results

Behavioral data collected during scanning indicates that patients showed impaired performance under all conditions (OrderedSilent, OrderedNoise, RandomSilent, RandomNoise) (Table 1, Table 2). A significant main effect of group was observed for errors of commission (F(3,36) = 17.3, p<0.001), and a trend towards a main effect of group was observed for errors of omission (F(3,36) = 2.95, p = 0.09). A significant condition X group interaction was observed for reaction time (F(3,36) = 4.71, p<0.01); this effect was driven by longer reaction times during the Ordered SART in Noise in patients compared to controls (p = 0.027, Fisher's LSD). Significant interactions were not observed for errors of commission (F(3,36) = 0.91, p = 0.45) or omission (F(3,36) = 0.85, p = 0.47).

**Table 1:** Behavioral Data, Ordered SART.

Measure	Group	Ordered Silent	Ordered Noise
% Errors of Commission	Control	4.59±1.17	3.94±1.20
	Patient	17.1±3.33	18.3±4.27
% Errors of Omission	Control	4.10±1.89	3.48±1.60
	Patient	7.64±1.88	7.87±1.93
Reaction Time (ms)	ControlPatient	291±14.3339±21.1	282±15.6342±21.3

±symbols represent the standard error of the mean.

**Table 2:** Behavioral Data, Random SART.

Measure	Group	Random Silent	Random Noise
% Errors of Commission	Control	21.9±3.70	23.2±4.21
	Patient	44.1±5.04	41.1±5.46
% Errors of Omission	Control	1.09±0.96	0.92±0.60
	Patient	3.05±0.86	2.27±0.56
Reaction Time (ms)	ControlPatient	368±15.4373±15.1	364±14.1377±14.3

± symbols represent the standard error of the mean.

### fMRI Results

**Figure 1:**
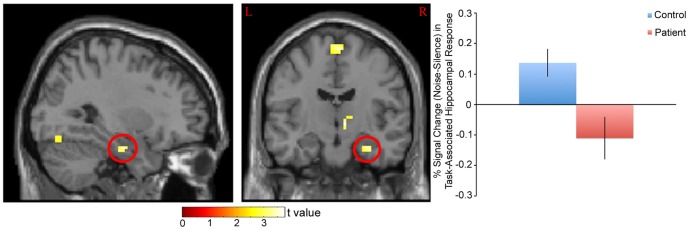
% Signal Change (Noise-Silence) in Task-Associated Right Hippocampal Response in Patients and Controls. *Left*: Statistical parametric map. Map was thresholded at p<0.01 and overlaid onto the SPM8 canonical single subject T1 image for visualization. Data are shown in the neurologic convention (R on R). *Right*: Extracted right hippocampus response, based on the cluster circled in red on the parametric map (peak coordinate: x = 30, y = −13, z = −23). A relative increase in task-associated response in noise (compared to silence) in controls and a decrease in response in patients was observed.

**Figure 2:**
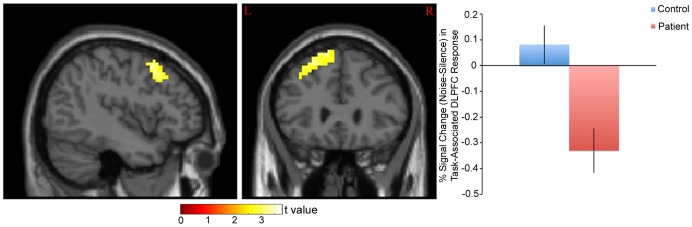
% Signal Change (Noise-Silence) in Task-Associated Left DLPFC Response in Patients and Controls. *Left*: Statistical parametric map. Map was thresholded at p<0.01 and overlaid onto the SPM8 canonical single subject T1 image for visualization. Data are shown in the neurologic convention (R on R). *Right*: Extracted left DLPFC response (peak coordinate: x = −39, y = 20, z = 40). A relative increase in task-associated response in noise (compared to silence) in controls and a decrease in response in patients was observed.

**Figure 3:**
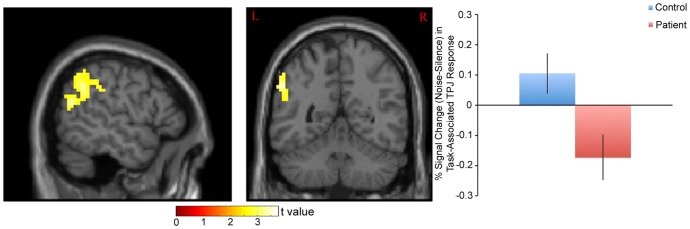
% Signal Change (Noise-Silence) in Task-Associated Left TPJ Response in Patients and Controls. *Left*: Statistical parametric map. Map was thresholded at p<0.01 and overlaid onto the SPM8 canonical single subject T1 image for visualization. Data are shown in the neurologic convention (R on R). *Right*: Extracted left TPJ response (peak coordinate: x = −57, y = −46, z = 28). A relative increase in task-associated response in noise (compared to silence) in controls and a decrease in response in patients was observed.

**Figure 4:**
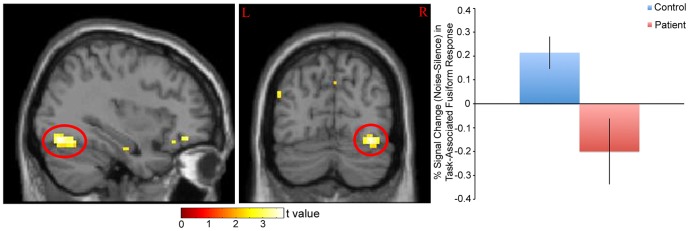
% Signal Change (Noise-Silence) in Task-Associated Right Fusiform Response in Patients and Controls. *Left*: Statistical parametric map. Map was thresholded at p<0.01 and overlaid onto the SPM8 canonical single subject T1 image for visualization. Data are shown in the neurologic convention (R on R). *Right*: Extracted right fusiform response, based on the cluster circled in red on the parametric map (peak coordinate: x = 39, y = −76, z = −14). A relative increase in task-associated response in noise (compared to silence) in controls and a decrease in response in patients was observed.

For this study, “task” associated response was defined as the effect of difficulty (Random – Ordered SART) on BOLD signal (See Methods). Based on this measure, a significant distraction (noise or silence) X diagnosis (patient or control) interaction was observed on task-associated response in the right hippocampus (peak coordinate x = 30, y = −13, z = −23; t = 3.53, p = 0.013; cluster size 69 voxels; Figure 1), left DLPFC (peak coordinate x = −39, y = 20, z = 40; t = 3.52, p = 0.014; cluster size 637 voxels; Figure 2), left TPJ (peak coordinate x = −57, y = −46, z = 28; t = 3.01, p = 0.050; cluster size 259 voxels; Figure 3), and right fusiform gyrus (peak coordinate x = 39, y = −76, z = −14; t = 3.55, p = 0.013; cluster size 65 voxels; Figure 4).

When reaction time was used as a covariate, response differences between groups remained significant for all four regions (right hippocampus (t = 4.07, p<0.01), left DLPFC (t = 3.51, p = 0.015), left TPJ (t = 3.16, p = 0.038), right fusiform gyrus (t = 3.57, p = 0.013)).

To further investigate how noise-associated processing contributed to the interaction effect, we compared the effect of noise under easy (Ordered) conditions between patients and controls in the hippocampus. Relative to controls, patients showed a trend towards increased hippocampal response during noise (relative to silence) under easy conditions (peak coordinate: x = 30, y = −13, z = −23; t = 2.69, p = 0.10, cluster size 23 voxels).

### Behavioral Correlates

**Figure 5:**
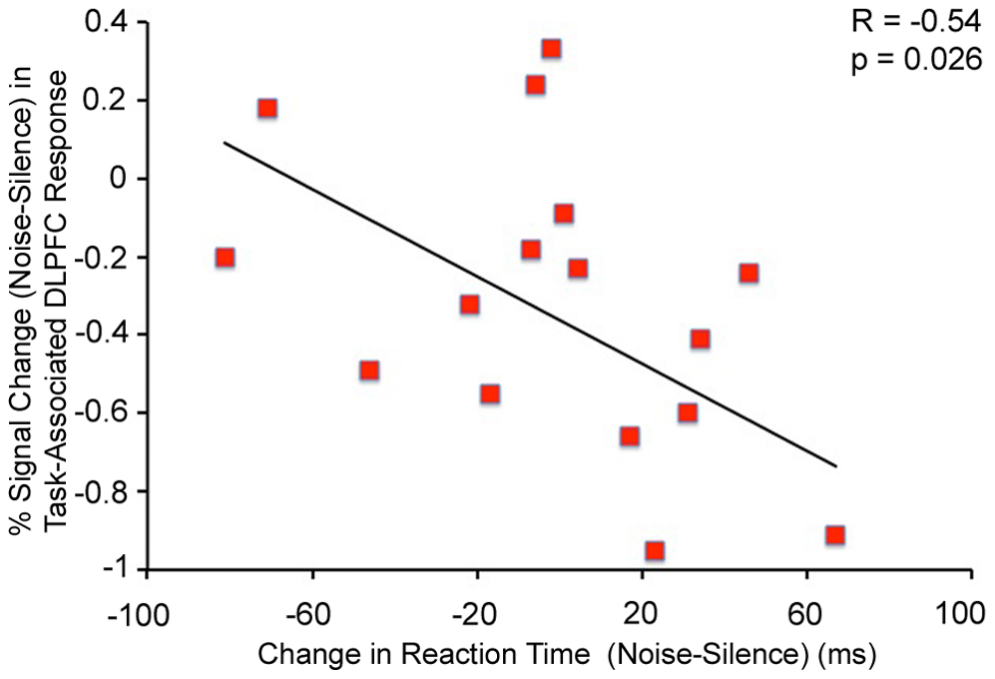
Negative Correlation between % Signal Change (Noise-Silence) in Task-Associated Left DLPFC Response and the Effect of Noise on Reaction Time during the Task in Patients.

Noise-induced recruitment of the left DLPFC during the task was negatively correlated with the effect of noise on reaction time in patients (F(1,15) = 6.09, R = −0.54, p = 0.026, Figure 5). A significant relationship was not observed in controls (F(1,21) = 2.06, R = −0.30, p = 0.17).

### Clinical Correlates

**Figure 6:**
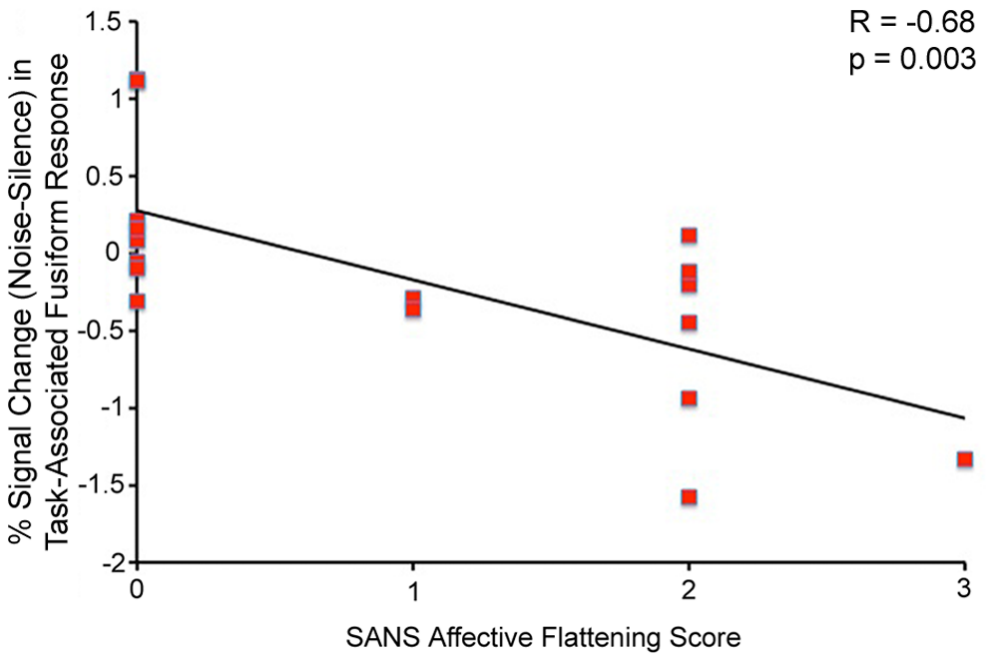
Negative Correlation between % Signal Change (Noise-Silence) in Task-Associated Right Fusiform Response and Patient SANS Affective Flattening Score.

**Figure 7:**
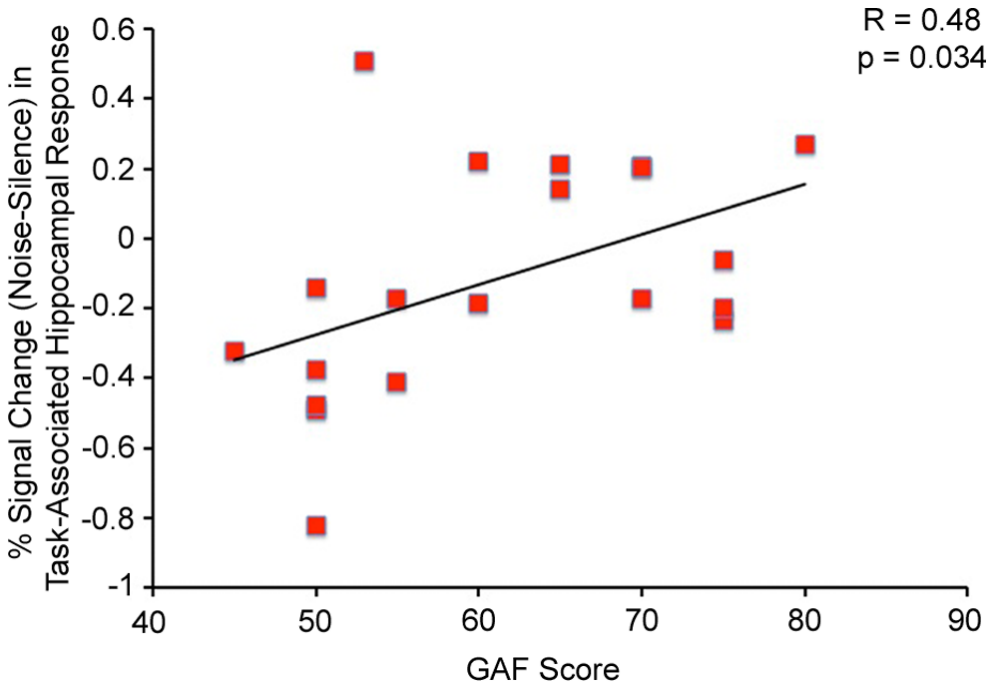
Positive Correlation between % Signal Change (Noise-Silence) in Task-Associated Right Hippocampal Response and Patient GAF Score.

Noise-induced recruitment of the right fusiform gyrus was negatively correlated with patient SANS Affective Flattening score (F(1,15)) = 12.89, R = −0.68, p = 0.003, Figure 6). A t-test comparing affected patients (SANS Affective Flattening scores of 1–3) with unaffected patients (SANS Affective Flattening score of 0) also revealed significantly lower noise-induced recruitment of this area in the affected patients (t = 3.12, p = 0.007). Noise-induced recruitment of the right hippocampus during the task was correlated with patient GAF score (F(1,18) = 5.25, R = 0.48, p = 0.034, Figure 7).

Behaviorally, a nearly-significant negative correlation was observed between noise-induced increase in reaction time during the task and GAF score (F(1,14) = 4.19, R = 0.48, p = 0.060). No significant correlations were observed between behavioral or fMRI measures and BPRS scores.

## Discussion

The primary findings of the present study were 1) noise differentially affected task-associated response in the hippocampus, DLPFC, TPJ, and fusiform gyrus in patients vs. controls, 2) noise-induced recruitment of the left DLPFC during the task was negatively correlated with the effect of noise on reaction time in patients, 3) noise-induced recruitment of the right fusiform gyrus during the task was negatively correlated with patient SANS affective flattening score, and 4) noise-induced recruitment of the right hippocampus during the task was correlated with patient GAF score. These results suggest that neural systems in schizophrenia are abnormally modulated during noise distraction, and that the magnitude of this difference may predict cognitive, social, occupational and psychological functioning.

The present study used a selective attention task that required the ability to detect rare events while filtering irrelevant noise. Based on previous fMRI studies that used similar task elements [4], [18], [19], [25]–[27], we examined task-related neural response in four regions, the hippocampus, DLPFC, TPJ, and fusiform gyrus. As hypothesized, relative to patients, controls showed greater neural response in all four regions when performing the task during noise (as compared to silence). The potential meaning of this differential response in respect to the function of each of these four regions is discussed in the following sections.

### Hippocampus

The hippocampus is a highly interconnected brain area that is involved in many cognitive functions, including episodic memory [28], spatial memory and navigation [29], attention-related processing [30], and sensory filtering [31]. Most relevant to the latter two functions, a characteristic feature of the hippocampus is that it shows suppressed response during repetitive auditory stimulation, likely due to recurrent feedback from inhibitory interneurons onto excitatory pyramidal cells [32]. Possibly as a result of this circuitry, the hippocampus has been hypothesized to play a role in mediating the relative balance between the strength of top-down (internally-driven) and bottom-up (stimulus-driven) inputs in functional neural representations [33], and may effectively help “gate” information going into areas with which it is connected (e.g. the DLPFC) [34]. Thus, sounds that are unlikely to be important (e.g. the incessant ticking of a clock) are not considered salient and consequently not consciously processed; however, stimuli that are likely to be important (e.g. stimuli essential for a task) are considered salient and more fully processed.

In regards to the present study, when distracting noise is overlaid on top of a cognitively engaging task (e.g. the Random SART), increased engagement of the hippocampus may occur in order to facilitate active, conscious processing of task-relevant stimuli (i.e. the numbers on the screen). The finding that patients are less able to recruit the hippocampus in noise as task difficulty is increased suggests that patients are less able to engage this area during noise distraction and is conceptually in agreement with other fMRI studies that have reported hippocampal hypoactivation in schizophrenia during cognitive tasks [25], [26], [35]. Although task-associated hypoactivation during noise in patients may seem counterintuitive due to previous reports of sensory flooding and hippocampal hyperactivity during passive listening [4], a reasonable interpretation is that during non-demanding tasks, noise distraction taxes the hippocampus near maximal capacity, occluding further recruitment as task difficulty in increased (relative to controls). A similar relationship between cognitive load and brain response has been observed using working memory tasks [36].

To test this hypothesis, recruitment of the hippocampus in noise (relative to silence) under easy conditions was compared in patients and controls. A trend towards hippocampal hyperactivity in noise under easy conditions (Ordered SART) was observed in patients. Although this finding is significant only at the trend level and therefore should be viewed cautiously, hippocampal hyperactivity is consistent with previous observations in schizophrenia patients using other tasks that are minimally taxing to cognitive systems, including fixation on a point [12], passive watching of fearful faces [37], smooth pursuit eye movement [13], and resting state (absence of a task) [14]. In regards to auditory stimulation, increased hippocampal response in patients during passive listening has been observed using the same urban noise stimulus as in the present study [4]. Together with a previous paper that observed a similar effect using repeated clicks [3], these results suggest that under cognitively non-demanding conditions, the hippocampus may be hyper-responsive to noise stimulation in schizophrenia. This phenotype is hypothesized to reflect hippocampal inhibitory dysfunction in schizophrenia that may underlie symptom etiology (e.g. sensory “flooding”) [38]. Altogether, increased sensitivity of the hippocampus during auditory stimulation as well as during cognitively non-demanding tasks may have deleterious consequences as task difficulty is increased, although the relative contribution of hyperactivity during low-load conditions to the interaction effect deserves further scrutiny in future studies.

### DLPFC

Perhaps even more so than the hippocampus, the DLPFC is an extraordinarily well-connected area that is involved in a multitude of cognitive functions. One of these functions is to orchestrate “proactive” cognitive control, including anticipatory prevention of interference from task-irrelevant stimuli (e.g. distracting noise) [39]. The DLPFC is able to achieve this function through “context” processing, defined as the neural representation of internal goals generated from prior knowledge that biases the selection of behavioral responses [39]. In terms of the present study, recruitment of the DLPFC during noise as task difficulty is increased may reflect the recruitment of voluntary, “top-down” filtering processes to facilitate processing of task-relevant stimuli. A relative decrease in patients (compared to controls) during noise suggests that patients are less able to recruit these processes to enhance performance. Consistent with this idea, the effect of noise on DLPFC response was correlated with its effect on reaction time in patients, suggesting that reduced activity in this region is associated with reduced processing speed, loss of focus, or other generalized dysfunction in task-related processing during noise distraction. The direction of this association (i.e. if reduced DLPFC activity causes distractibility, or if distractibility causes reduced DLPFC activity) cannot be determined by the present study. It is also possible that hippocampal dysfunction in patients contributes to the observed functional abnormalities in the DLPFC, given the extensive connectivity between these two areas [34].

### TPJ

As its name suggests, the temporoparietal junction is located at the border of the temporal and parietal cortex, in the posterior region of the brain. This area is part of a ventral attention network that is involved in processing task-relevant, salient stimuli, particularly when these stimuli are infrequent [40]–[46]. For example, increased bilateral TPJ activation has been observed when auditory or visual stimuli change configuration (e.g. are rotated, change pitch, etc.), but only when subjects are instructed to attend to stimulus changes [41]. TPJ activation is thus largely “bottom-up” in nature in that it is driven by sensory activity. In regards to the present study, increased TPJ response during the task in noise may thus reflect a cognitive load-driven increase in processing resources to facilitate detection of the irregular “no-go” stimuli (the number “3”). These processes may be particularly important when stimuli are unpredictable (as during the Random SART) compared to when they are predictable (as during the Ordered SART). Decreased TPJ recruitment in patients (relative to controls) may therefore reflect the relative inability to engage “bottom-up” processes during task performance. Relative hypoactivation of the TPJ during the task is consistent with previous fMRI studies that have examined the neural correlates of deviant stimulus detection in schizophrenia [25], [26].

Differences in TPJ recruitment between controls and patients were primarily left-lateralized, in contrast to a previous study which showed right-lateralized TPJ effects in schizophrenia during an auditory tone discrimination task with distracting noise [15]. In addition, although numerous previous studies have implicated the TPJ in cognitive control, many of these effects have been right-lateralized [47]. Recent studies, however, have indicated specific roles for the left TPJ in cognitive processing. These roles may include functional integration of “bottom-up” (i.e. sensory stimulus-driven) processing with “top-down” processing [48], as well as specialization for verbal (as opposed to spatial) information processing [49]. The finding that both the right and left TPJ may show task-related deficits in schizophrenia suggests that dysfunction of this region is not hemisphere-specific in the illness and rather may depend on task conditions. Additional studies that examine recruitment of this area under different task conditions will be needed to determine the specific roles of the left and right TPJ in visual attention.

### Fusiform Gyrus

The fusiform gyrus is a large (50 mm in length – equal to the distance from V1 to V5 of visual cortex) [50] ventral posterior cortical area involved in higher level visual processing. The fusiform is active during many types of visual tasks, including face processing [50], reading and language [51], and object recognition [52]. Evidence suggests that the fusiform is divided into anatomically and functionally unique subregions that specialize in various aspects of processing visual information (e.g. the fusiform face area [53] and the visual word form area [54]). In regards to the present study, recruitment of the fusiform gyrus likely represents an increase in cortical resources devoted to processing visual number stimuli. The right posterior location of the effect is in agreement with a previous study that showed preferential recruitment of the right posterior fusiform for numbers [55]. Relative fusiform hypoactivation in patients (relative to controls) suggests that patients are less able to engage this area during auditory distraction, and further implies that sensory processing deficits in schizophrenia may induce cross-modal functional abnormalities. Fusiform dysfunction is consistent with previous studies in patients, although both hypoactivation [56], [57] and hyperactivation [13], [58], [59] has been observed. The directionality of these effects may be dependent on the nature of the task (e.g. task difficulty).

### Clinical Correlates

The ability to recruit the right fusiform gyrus was negatively correlated with SANS Affective Flattening score in patients. This score describes a subject's outward display of emotions, such as gestures, tone of voice, eye contact, facial expressions, and appropriate laughter or smiling [60]. Higher scores imply less emotional affect and are classified as negative symptoms of schizophrenia. In agreement with a role for fusiform dysfunction in negative symptoms, a previous study found an association between negative symptoms and lower metabolic rate in this area [61]. In addition, reduced right posterior fusiform gray matter is associated with reduced extraversion in patients, suggesting that dysfunction in this region may contribute to illness-related social disturbances [62].

The ability to recruit the hippocampus during noise as task difficulty was increased was correlated with patient GAF score. The GAF scale provides a measurement of social, occupational, and psychological functioning; higher functioning patients have higher scores [63]. Given that patients were less able to recruit the hippocampus during noise than controls, this result suggests that the severity of hippocampal dysfunction may predict lower functioning in schizophrenia. Previous studies have observed negative associations between GAF scores and sensory processing impairments, including deficits in auditory gating [20] and generation of the mismatch negativity [64]. In addition, preliminary results have reported that auditory training improves sensory gating as well as verbal learning and memory deficits in patients [65], [66]. These findings suggest that modulation of sensory processing may have therapeutic efficacy in the illness.

### Conclusion

This study found that relative to healthy controls, patients with schizophrenia showed abnormalities in noise-induced neural response during a visual attention task. These changes were associated with higher SANS affective flattening subscore and lower global functioning, suggesting that functional impairment may contribute to clinical symptoms of schizophrenia and be associated with reduced quality of life. This work is the first to demonstrate that previously reported auditory processing abnormalities [4] may be associated with neural response changes during cross-modal, visual attention tasks in schizophrenia.
